# Cardiovascular Adverse Events Associated With BRAF and MEK Inhibitors: A Systematic Review and Meta-analysis

**DOI:** 10.1001/jamanetworkopen.2019.8890

**Published:** 2019-08-09

**Authors:** Raluca I. Mincu, Amir A. Mahabadi, Lars Michel, Simone M. Mrotzek, Dirk Schadendorf, Tienush Rassaf, Matthias Totzeck

**Affiliations:** 1 West German Heart and Vascular Center, Department of Cardiology and Vascular Medicine, University Hospital Essen, Essen, Germany; 2 West German Cancer Center, Department of Dermatology, University Hospital Essen, Essen, Germany

## Abstract

**Importance:**

Cardiovascular adverse events (CVAEs) after treatment with BRAF and MEK inhibitors in patients with melanoma remain incompletely characterized.

**Objective:**

To determine the association of BRAF and MEK inhibitor treatment with CVAEs in patients with melanoma compared with BRAF inhibitor monotherapy.

**Data Sources:**

PubMed, Cochrane, and Web of Science were systematically searched for keywords *vemurafenib*, *dabrafenib*, *encorafenib*, *trametinib*, *binimetinib*, and *cobinimetinib* from database inception through November 30, 2018.

**Study Selection:**

Randomized clinical trials reporting on CVAEs in patients with melanoma being treated with BRAF and MEK inhibitors compared with patients with melanoma being treated with BRAF inhibitor monotherapy were selected.

**Data Extraction and Synthesis:**

Data assessment followed the Preferred Reporting Items for Systematic Reviews and Meta-analysis (PRISMA) guidelines. Pooled relative risks (RRs) and 95% CIs were determined using random-effects and fixed-effects analyses. Subgroup analyses were conducted to assess study-level characteristics associated with CVAEs.

**Main Outcomes and Measures:**

The selected end points were pulmonary embolism, a decrease in left ventricular ejection fraction, arterial hypertension, myocardial infarction, atrial fibrillation, and QTc interval prolongation. All-grade and high-grade (≥3) CVAEs were recorded.

**Results:**

Overall, 5 randomized clinical trials including 2317 patients with melanoma were selected. Treatment with BRAF and MEK inhibitors was associated with an increased risk of pulmonary embolism (RR, 4.36; 95% CI, 1.23-15.44; *P* = .02), a decrease in left ventricular ejection fraction (RR, 3.72; 95% CI, 1.74-7.94; *P* < .001), and arterial hypertension (RR, 1.49; 95% CI, 1.12-1.97; *P* = .005) compared with BRAF inhibitor monotherapy. The RRs for myocardial infarction, atrial fibrillation, and QTc prolongation were similar between the groups. These results were consistent when assessing high-grade CVAEs (left ventricular ejection fraction: RR, 2.79; 95% CI, 1.36-5.73; *P* = .005; *I*^2^ = 29%; high-grade arterial hypertension: RR, 1.54; 95% CI, 1.14-2.08; *P* = .005; *I*^2^ = 0%), but RRs for high-grade pulmonary embolism were similar between groups. A higher risk of a decrease in left ventricular ejection fraction was associated with patients with a mean age younger than 55 years (RR, 26.50; 95% CI, 3.58-196.10; *P* = .001), and the associated risk of pulmonary embolism was higher for patients with a mean follow-up time longer than 15 months (RR, 7.70; 95% CI, 1.40-42.12; *P* = .02).

**Conclusions and Relevance:**

Therapy with BRAF and MEK inhibitors was associated with a higher risk of CVAEs compared with BRAF inhibitor monotherapy. The findings may help to balance between beneficial melanoma treatment and cardiovascular morbidity and mortality.

## Key Points

**Question:**

What is the rate of cardiovascular adverse events among patients with melanoma treated with BRAF and MEK inhibitors compared with patients treated with BRAF inhibitor monotherapy?

**Findings:**

In this systematic review and meta-analysis of 5 randomized clinical trials including 2317 patients, treatment with BRAF and MEK inhibitors was associated with a higher risk of pulmonary embolism, decrease in left ventricular ejection fraction, and arterial hypertension compared with treatment with BRAF inhibitor monotherapy. The risks of myocardial infarction, atrial fibrillation, and QTc prolongation were similar between groups.

**Meaning:**

These findings demonstrate an association of cardiovascular adverse events with BRAF and MEK inhibitor therapy, which may guide clinical cardio-oncological management.

## Teaser

This systematic review and meta-analysis determines the association of BRAF and MEK inhibitor treatment with cardiovascular adverse events in patients with melanoma compared with BRAF inhibitor monotherapy.

## Introduction

The incidence of melanoma has been increasing, and it now accounts for 55 500 cancer deaths annually worldwide.^1^ The most frequent mutations in melanoma occur at the level of BRAF with a subsequent upregulation of the canonical MAPK pathway responsible for tumor growth and proliferation.^2,3^ The identification of *BRAF* mutations prompted the development of a new class of targeted cancer drugs: BRAF inhibitors. Subsequent research showed that treatment with BRAF inhibitors alone causes resistance through a paradoxical signaling cascade mediated by MEK, leading to the development of MEK inhibitor therapies.^1,4,5^ The combination of BRAF and MEK inhibitor therapy has emerged as an optimal treatment of metastatic *BRAF*-mutated melanoma, with improved survival rates compared with monotherapy.^6,7,8,9,10^ A 2018 study^11^ found that the first BRAF and MEK inhibitor combination showed significant improvement in relapse-free survival in treating adjuvant stage III melanoma, which led to global approval. To date, 3 BRAF inhibitors (dabrafenib,^12^ vemurafenib,^13^ and encorafenib^7^) and 3 MEK inhibitors (trametinib,^14^ cobinimetinib,^15^ and binimetinib^16^) have received US Food and Drug Administration and European Medicines Agency approval. Common combination therapies are dabrafenib and trametinib,^9^ vemurafenib and cobimetinib,^15^ and encorafenib and binimetinib.^17^

Cardiovascular adverse events (CVAEs) associated with BRAF and MEK inhibitors have been reported in several studies, particularly regarding a reduction in left ventricular ejection fraction (LVEF), arterial hypertension, and prolongation of QTc interval. However, data suggest that the spectrum of CVAEs is broader and could have tremendous effects on maintaining these patients’ antitumoral treatment.^18^

The inhibition of BRAF and MEK negatively interferes with cardiovascular MAPK signaling. This generates oxidative stress and apoptosis of myocytes and impairs angiogenesis, leading to significant cardiovascular diseases.^19,20,21,22^ Clinical data document a reduction in LVEF under BRAF and MEK inhibitors in 5% to 11% of all patients, while arterial hypertension occurred in 10% to 15% of patients.^10,17^ Although QTc interval prolongation is denoted as a potential CVAE, several studies reported no association of BRAF and MEK inhibitor therapy with QTc intervals.^10,17^ Therefore, questions remain about whether this is a salient CVAE.

The nature and incidence of CVAEs associated with BRAF and MEK inhibitor therapy are incompletely described. However, cardiovascular complications may affect a patient’s quality of life or may require temporary or permanent cancer therapy termination. With this systematic review and meta-analysis, we set out to clarify the type, incidence, and relative risk of CVAEs in patients with melanoma who are being treated with a combination of BRAF and MEK inhibitors compared with patients receiving BRAF inhibitor monotherapy.

## Methods

This meta-analysis was performed in accordance with the Preferred Reporting Items for Systematic Reviews and Meta-analyses (PRISMA) reporting guideline^23^ and followed the Cochrane Handbook for Systematic Reviews of Interventions recommendations.^24^ The study was registered with PROSPERO (CRD42018108198). A systematic search was conducted through PubMed, Cochrane, and Web of Science databases, the major cardiology websites (TCTMD, Clinical Trial Results, Medscape, and CardioSource Plus), and abstracts or presentations from annual meetings of the major cardiovascular and cancer societies to identify relevant studies published from the inception of the databases to November 30, 2018, using the search terms *vemurafenib*, *dabrafenib*, *encorafenib*, *trametinib*, *binimetinib*, and *cobinimetinib*. We made our search specific and sensitive using Medical Subject Heading terms and free text and considered studies published in English only. The search strategy through PubMed is depicted in eTable 1 in the Supplement.

Because the purpose of this study was to summarize the incidence of overall and high-grade CVAEs in patients with melanoma receiving BRAF and MEK inhibitors, we restricted our study to randomized clinical trials (RCTs) in which adult participants received the available combinations of BRAF inhibitors and MEK inhibitors (ie, dabrafenib and trametanib, vemurafenib and cobimetinib, or encorafenib and binimetinib) and were randomly assigned to a treatment or a control group. The meta-analysis excluded abstracts, reviews, animal and in vitro studies, meta-analyses, case reports, single-arm BRAF and MEK inhibitor treatment studies, monotherapy with BRAF inhibitor studies, studies with MEK inhibitor treatment with other therapies for melanoma, nonrandomized clinical trials, studies that did not report on CVAEs, and special population studies (eg, elderly population, population from a certain geographic region, pediatric population).

After removing duplicates, 3 of us (R.I.M., L.M., and S.M.M.) independently reviewed the abstracts. Any discrepancies in results between the 3 investigators were solved by discussion with the other investigators. When the inclusion criteria appeared to be met, the full-text publication was reviewed by the 3 authors mentioned above. At the end of the review process, the full texts of the studies considered eligible were reviewed by all investigators.

### Data Extraction and Quality Assessment

Three of us (R.I.M., L.M., and S.M.M.) independently performed data extraction using a standard data extraction form that contained the following fields: (1) publication details (ie, name of the first author and year of publication), (2) study design, (3) characteristics of study population (ie, sample size, age and sex distribution), (4) therapy dosage, (5) mean follow-up, and (6) study end points. Study-level characteristics were abstracted. The trial quality was assessed by 3 of us (R.I.M., L.M., and S.M.M.) for each study separately against the following criteria according to the Cochrane Risk-of-Bias Tool^24^: (1) random sequence generation (ie, selection bias), (2) allocation concealment (ie, selection bias), (3) blinding of participants and personnel (ie, performance bias), (4) blinding of outcome assessment (ie, detection bias), (5) incomplete outcome data, (6) selective reporting (ie, reporting bias), and (7) other bias (ie, measurement error, observer variability, dose of drug, length of follow-up, and characteristics of participants). Authors resolved disagreement by consensus, and a fourth author (M.T.) was consulted to resolve disagreement.

### Study End Points

The end points were defined according to the National Cancer Institute Common Terminology Criteria for Adverse Events version 4.^25^ All-grade and high-grade (ie, grade 3-5, indicating severe, life threatening, or causing death) treatment-emergent CVAEs were abstracted. The selected end points were as follows: (1) pulmonary embolism, (2) a decrease in LVEF, (3) arterial hypertension, (4) myocardial infarction, (5) atrial fibrillation, and (6) QTc interval prolongation. The definitions of study end points are detailed in eTable 2 in the Supplement.

### Statistical Analysis

The meta-analysis was conducted on eligible studies by dividing the patients into the following 2 groups: (1) the BRAF and MEK inhibitor group, which included patients with melanoma treated with a combination of BRAF inhibitors and MEK inhibitors; and (2) the control group, which included patients with melanoma treated with BRAF inhibitor monotherapy. The proportion of patients with CVAEs receiving BRAF and MEK inhibitors was compared with that of the control group in the same RCT. All studies included in our meta-analysis reported the number of patients with adverse events and not the number of adverse events for a group. The data are expressed as percentage of patients with CVAEs, calculated by dividing the number of each CVAE by the total sample size. Risk ratios (RRs) and 95% CIs are used to express dichotomous outcomes.^26^ Statistical significance was set at *P* < .05, and all tests were 2-tailed. A 2018 study^17^ had 3 arms: an encorafenib and binimentinib arm, an encorafenib arm, and a vemurafenib arm. We compared the patients with adverse events from the encorafenib and binimetinib arm with those from the encorafenib arm and the patients with adverse events from the encorafenib and binimentinib arm with those from the vemurafenib arm. For the analysis, we used both random-effects and fixed-effects models. A random-effects model was preferred owing to the assumption that different studies are estimating different yet related intervention effects. In the presence of heterogeneity, the use of the random-effects method will result in wider CIs for the average intervention and corresponding claims of statistical significance will be more conservative.^24^ We considered both the presence of a *P* value at 10% of the level of significance (*P* < .01) derived from Q analysis and a value of inconsistency (*I*^2^) of more than 40% to denote heterogeneity between the selected studies. A value of *I*^2^ less than 40% denoted that heterogeneity might not be important, *I*^2^ from 40% to 60% may have represented moderate heterogeneity, *I*^2^ from 50% to 90% may have represented substantial heterogeneity, and *I*^2^ from 75% to 100% represented considerable heterogeneity.^24^ The funnel plot test could not be used to assess publication bias because our analysis included fewer than 10 studies.^24^ The analyses were conducted using Review Manager version 5.3 (The Cochrane Collaboration) and the Comprehensive Meta-Analysis (Biostat, Inc).

## Results

### Eligible Studies and Characteristics

**Figure 1.**
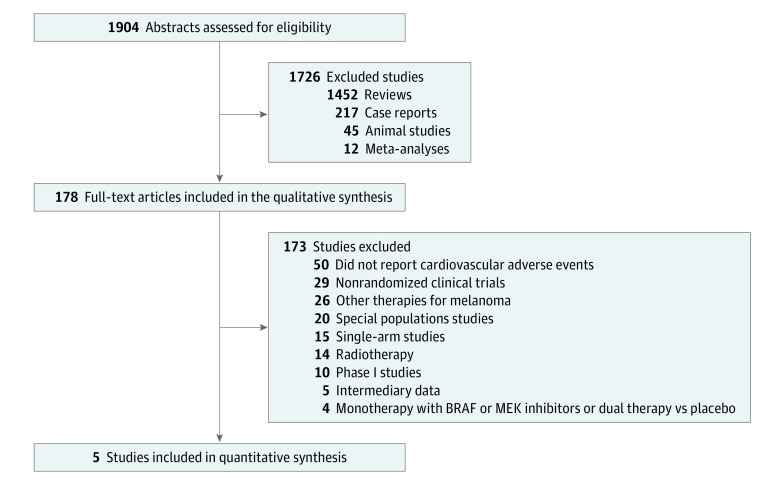
PRISMA Selection Flowchart

A total of 1904 eligible articles were identified through database searching (Figure 1). We identified 5 RCTs of patients receiving BRAF and MEK inhibitor therapy compared with patients receiving BRAF inhibitor monotherapy.^6,9,10,17,27^ A total of 2317 patients with melanoma were included. The agreement between the authors that performed the search was more than 88%, with a κ of more than 83.3%. General characteristics of the study population are detailed in Table 1.

**Table 1.** Characteristics of the Studies

Source	Acronym	Type	Cancer Type	Treatment	No. of Patients	Age, Mean (Range), y	Men, No. (%)	BRAF and MEK Inhibitor Dosage	Follow-up, Median (IQR), mo
Flaherty et al,^27^ 2012	NA	RCT II	Metastatic melanoma and *BRAF* V600 mutations	Dabrafenib and trametinib	54	58 (27-79)	34 (63.0)	Dabrafenib 150 mg twice daily and trametinib 2 mg once daily	14.1 (10.8-17.6)
				Dabrafenib and placebo	54	50 (18-52)	29 (53.7)	Dabrafenib 150 mg twice daily	
Robert et al,^6^ 2015	COMBI-v	RCT III	Unresectable stage IIIC or IV melanoma with *BRAF* V600 mutations	Dabrafenib and trametinib	352	55 (18-51)	208 (59.1)	Dabrafenib 150 mg twice daily and trametinib 2 mg once daily	10 (NA)
				Vemurafenib	352	54 (NA)	180 (51.1)	Vemurafenib 960 mg twice daily	11 (NA)
Ascierto et al,^10^ 2016	coBRIM	RCT III	Unresectable stage IIIC or stage IV melanoma with *BRAF* V600 mutations	Vemurafenib and cobimetinib	247	56 (23-88)	146 (59.1)	Vemurafenib 960 mg twice daily and cobimetinib 60 mg once daily	14.2 (8.5-17.3)
				Vemurafenib and placebo	248	55 (25-85)	140 (56.5)	Vemurafenib 960 mg twice daily	
Long et al,^9^ 2017	COMBI-d	RCT III	Unresectable stage IIIC or stage IV melanoma with *BRAF* V600 mutation	Dabrafenib and trametinib	211	55 (22-89)	111 (52.6)	Dabrafenib 150 mg twice daily and trametinib 2 mg once daily	>36 (NA)
				Dabrafenib and placebo	212	57 (22-86)	114 (53.7)	Dabrafenib 150 mg twice daily	
Dummer et al,^17^ 2018	COLUMBUS	RCT III	Unresectable stage stage IIIB, IIIC, or IV, with *BRAF* V600 mutations	Encorafenib plus binimetinib	192	57 (20-89)	115 (59.9)	Encorafenib 450 mg once daily and binimetinib 45 mg twice daily	16.7 (16.3-18.4)
				Encorafenib	194	54 (23-88)	108 (55.7)	Encorafenib 300 mg once daily	16.6 (14.8-18.1)
				Vemurafenib	191	56 (21-82)	111 (58.1)	Vemurafenib 960 mg twice daily	14.4 (10.1-16.6)

Abbreviations: IQR, interquartile range; NA, not available; RCT, randomized clinical trial.

### Risk Ratios of CVAEs

**Figure 2.**
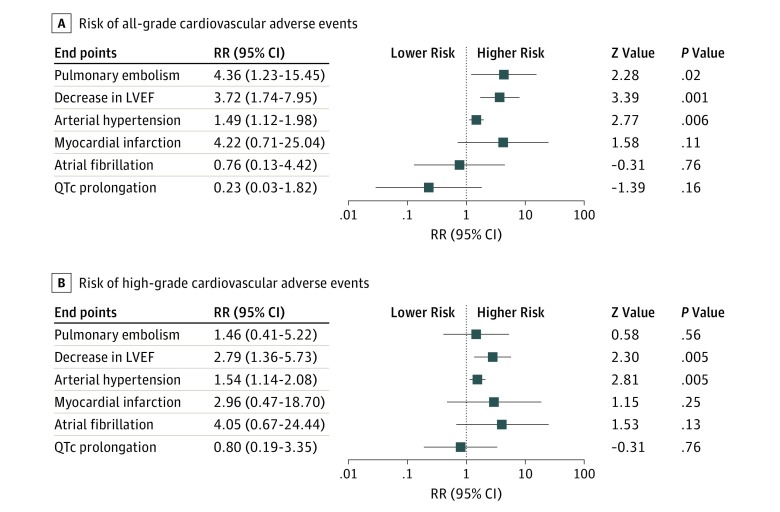
Overall Study Estimates of the Risk Ratio (RR) of Cardiovascular Adverse Events Associated With BRAF and MEK Inhibitor Treatment vs BRAF Inhibitor Monotherapy LVEF indicates left ventricular ejection fraction.

**Figure 3.**
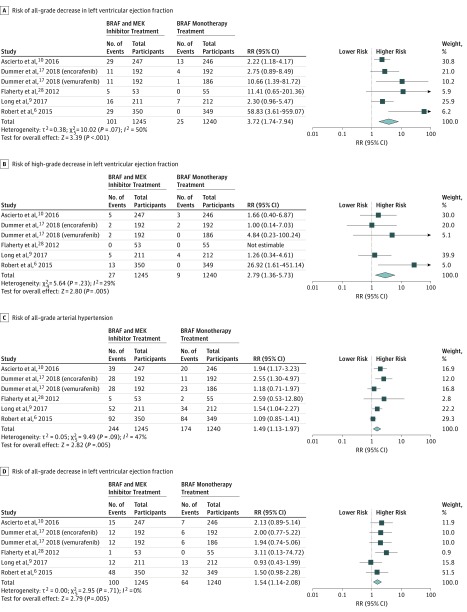
Overall and Individual Study Estimates of the Risk Ratio (RR) for a Decrease in Left Ventricular Ejection Fraction and Arterial Hypertension Associated With BRAF and MEK Inhibitor Treatment vs BRAF Inhibitor Monotherapy

The risk of all-grade CVAE calculated as RRs are depicted in Figure 2. Analysis revealed that therapy with BRAF and MEK inhibitors was associated with a risk of pulmonary embolism (RR, 4.36; 95% CI, 1.23-15.44; *P* = .02; *I*^2^ = 0%), with 2.2% of patients from the BRAF and MEK group developing pulmonary embolism compared with 0.4% of the BRAF inhibitor monotherapy group. The combination of BRAF and MEK inhibitors was associated with a more than 3-fold increase in risk of a decrease in LVEF and a 1.4-fold increase in risk of arterial hypertension (LVEF: RR, 3.72; 95% CI, 1.74-7.95; *P* < .001; *I*^2^ = 50%; arterial hypertension: RR, 1.49; 95% CI, 1.12-1.98; *P* = .005; *I*^2^ = 47%) (Figure 3). Overall, 8.1% of patients from the BRAF and MEK inhibitor treatment group had a decrease in LVEF compared with only 2.0% in the control group, and 19.5% of patients in the BRAF and MEK treatment group developed arterial hypertension, compared with 14.0% in the BRAF inhibitor control group. The RRs of myocardial infarction, atrial fibrillation, and QTc interval prolongation were similar between the BRAF and MEK inhibitor group and the BRAF inhibitor monotherapy control group (Figure 2).

### Subgroup Analysis

The high-grade end points, consisting of grade 3 to 5 CVAEs (ie, severe, life-threatening, or causing death) were analyzed (Figure 2). Treatment with BRAF and MEK inhibitors was associated with an increased risk of a high-grade decrease in LVEF (RR, 2.79; 95% CI, 1.36-5.73; *P* = .005; *I*^2^ = 29%) and high-grade arterial hypertension (RR, 1.54; 95% CI, 1.14-2.08; *P* = .005; *I*^2^ = 0%) (Figure 3). In contrast with the risk of all-grade pulmonary embolism, the risk of high-grade pulmonary embolism was similar between groups (Figure 2). The RR of high-grade myocardial infarction, atrial fibrillation, and QTc interval prolongation were similar between the BRAF and MEK inhibitor group and the control group (Figure 2). The proportion of patients from the dual treatment group who had a high-grade CVAE compared with the control group were 2.1% vs 0.7% for decrease in LVEF and 8.0% vs 5.1% for arterial hypertension. The analyses for all-grade and high-grade pulmonary embolism and myocardial infarction are depicted in eFigure 1 in the Supplement, and all-grade and high-grade atrial fibrillation and QTc interval prolongation are depicted in eFigure 2 in the Supplement.

A higher risk of decrease in LVEF was associated with patients with a mean age younger than 55 years (RR, 26.50; 95% CI, 3.58-196.10; *P* = .001), while the association with arterial hypertension was not significant for the younger population subgroup (RR, .016; 95% CI, 0.75-1.79; *P* = .50) (Table 2). The risk of pulmonary embolism was higher for patients with a mean follow-up time of more than 15 months (RR, 7.70; 95% CI, 1.40-42.12; *P* = .02). The study-level characteristics for the subgroup analysis are listed in eTable 3 in the Supplement.

**Table 2.** Risk Ratio of CVAEs Derived From Subgroup Analysis

Subgroup Analysis for CVAEs	Decrease in LVEF		Pulmonary Embolism		Atrial Fibrillation		Arterial Hypertension	
	RR (95% CI)	*P* Value	RR (95% CI)	*P* Value	RR (95% CI)	*P* Value	RR (95% CI)	*P* Value
Mean age, y								
≤55	26.50 (3.58-196.10)	.001	NA	NA	NA	NA	0.16 (0.75-1.79)	.50
>55	2.50 (1.59-3.94)	<.001	4.90 (1.23-19.58)	.02	0.76 (0.13-4.39)	.76	1.65 (1.24-2.18)	<.001
Mean follow-up, mo								
≤15	4.57 (2.59-8.04)	<.001	1.99 (0.18-21.82)	.57	2.99 (0.82-10.90)	.10	1.46 (0.88-2.43)	.14
>15	3.15 (1.66-5.98)	<.001	7.70 (1.40-42.12)	.02	0.28 (0.06-1.36)	.11	1.73 (1.13-2.64)	.01

Abbreviations: CVAE, cardiovascular adverse events; LVEF, left ventricular ejection fraction; NA, not available; RR, risk ratio.

### Heterogeneity and Sensitivity Analysis

The heterogeneity for each analysis of CVAEs was not statistically significant, except for decrease in LVEF, arterial hypertension, and QTc interval prolongation analyses, where heterogeneity could be rated as moderate. A sensitivity analysis was performed by excluding each study in a stepwise manner from the analysis to determine the relative importance of each study. Treatment with BRAF and MEK inhibitors remained a risk factor for the selected outcomes.

### Publication Bias Assessment

The studies were reviewed for publication bias. The risk of bias of the included studies was low, except for the blinding of outcome assessment, as depicted in eFigure 3 in the Supplement.

## Discussion

We performed a meta-analysis of CVAEs for patients with melanoma being treated with BRAF and MEK inhibitors using data from 5 RCTs with 2317 participants. The main results of our study were as follows: (1) BRAF and MEK inhibitor therapy was associated with a higher RR of pulmonary embolism, decrease in LVEF, and arterial hypertension compared with BRAF inhibitor monotherapy; (2) BRAF and MEK inhibitor therapy was not associated with higher rates of myocardial infarction, atrial fibrillation, or QTc interval prolongation compared with BRAF inhibitor monotherapy; (3) the RRs of high-grade decrease in LVEF and high-grade arterial hypertension were higher in the group being treated with BRAF and MEK inhibitors than in the group being treated with BRAF inhibitor monotherapy; (4) a higher risk of a decrease in LVEF was associated with patients with a mean age younger than 55 years; and (5) BRAF and MEK inhibitor therapy was associated with a higher risk of pulmonary embolism in studies with a mean follow-up time of more than 15 months.

The mechanisms of cardiotoxicity for BRAF and MEK inhibitors are incompletely understood. The occurrence of arterial hypertension could be triggered by interference with 2 mechanisms. One is the perturbance of the renin-angiotensin system regulation induced by the inhibition of BRAF and MEK signaling.^28^ The other mechanism is associated with the reduced bioavailability of nitric oxide. Nitric oxide production is reduced because of an impaired vascular endothelial growth factor pathway, mediated through the MAPK pathway.^29^ The upregulation of cluster of differentiation 47 induced by BRAF and MEK inhibition also inhibits the signaling of nitric oxide–cyclic guanosine monophosphate, reduces nitric oxide bioavailability, and thus contributes to recurrent vasoconstriction, hypertension, and an imbalance between thrombotic and antithrombotic states.^30,31^ This could serve as an explanation for the occurrence of pulmonary embolism and myocardial infarction.

Evidence for the pathomechanism underlying the association of BRAF and MEK inhibitor therapy with LVEF impairment is particularly scarce. Experimental evidence suggests that the MAPK pathway has a cardioprotective role in the heart. Treatment with BRAF and MEK inhibitors could lead to a perturbance of the physiological mechanisms of hypertrophy, apoptosis, remodeling of myocytes, and subsequently to a reduction in LVEF though this pathway.^19,22^ Another hypothesis is that an impairment of angiogenesis relies on the MAPK pathway to activate smooth muscle cell proliferation.^20^ This may contribute to hypertension, decreased LVEF, occurrence of atrial fibrillation, and, in the long term, the development of heart failure.

The decrease in LVEF seems to be a MEK-mediated adverse event. It was higher in the BRAF and MEK inhibitor arms compared with BRAF inhibitor monotherapy arms, concordant with the rates published in the individual studies included in the meta-analysis.^6,9,10,14,17^ A higher risk of a decrease in LVEF has been reported in other studies, probably owing to a lower number of included patients.^32,33^ An analysis concerning the proportion of patients with reduced ejection fraction who developed heart failure was not possible because only 2 studies^10,17^ reported a reduced number of events not tailored on the actual cardiological definition of heart failure.^34^ While QTc interval prolongation during the therapy with BRAF and MEK inhibitors was higher compared with control therapies, it was statistically insignificant, which is concordant with our findings.^33^ Prolonged QTc of more than 60 milliseconds compared with baseline or more than 500 milliseconds was associated with BRAF inhibitors vemurafenib^35^ and dabrafenib^36^ but not with the MEK inhibitor trametinib.^14,27^ To our knowledge, the RRs of pulmonary embolism, atrial fibrillation, and myocardial infarction were analyzed for the first time in our study.

It has been shown that the effective inhibition of the MAPK pathway through a combination of BRAF and MEK inhibitor therapy resulted in better clinical outcomes,^17^ and it is known to ameliorate BRAF inhibition–related adverse events caused by MEK hyperproduction, like squamous skin cancer and other skin-related toxicities.^6,37^ Although the available BRAF and MEK inhibitor combinations have largely overlapping adverse events, pyrexia has been associated with dabrafenib and trametinib^9^ and photosensitivity with vemurafenib and cobimetinib,^10^ but both adverse effects were infrequent with encorafenib and binimetinib.^17^ A direct comparison among the 3 combinations has not been performed until now.

However, it is difficult to interpret if the association with an increased risk of a decrease in LVEF and arterial hypertension under the BRAF and MEK combination presented here is a MEK-mediated adverse event or if this is subsequent to a more efficient inhibition of the MAPK pathway through dual therapy. A 2012 study^14^ comparing the MEK inhibitor trametinib with chemotherapy showed that 6.6% of patients in the MEK inhibitor group experienced decrease of LVEF with no reported cases of decrease in LVEF in the chemotherapy group. However, the difference between the groups did not reach statistical significance. On the other side, RCT trials of BRAF inhibitors did not report on a decrease in LVEF. Furthermore, the observed association of BRAF and MEK inhibitors with arterial hypertension has not been confirmed in studies of BRAF and MEK inhibitors compared with placebo^8^ or the MEK inhibitor trametinib compared with chemotherapy.^14^ On the other side, studies of the BRAF inhibitor vemurafenib compared with dacarbasine^38^ or with placebo^39^ showed a significant association with arterial hypertension. The increased rate of pulmonary embolism under dual therapy is not supported by a high rate of pulmonary embolism in patients treated with BRAF inhibitors alone vs chemotherapy^38^ or placebo.^39^

Our findings increase the awareness of CVAEs in patients with melanoma treated with BRAF and MEK inhibitors and may help to determine the appropriate clinical cardio-oncology management. The management of CVAEs is based on expert opinion, and the available guidelines do not give specific indications.^40^ A single clinical approach algorithm^41^ for the management of BRAF and MEK inhibitor–related CVAE has been already published. The clinical approach states that therapy with BRAF and MEK inhibitors should be stopped in case of stage 2 hypertension, defined as systolic blood pressure more than 160 mm Hg or diastolic blood pressure more than 100 mm Hg. Arterial hypertension should be treated according to current guidelines, and therapy should be reintroduced at a reduced dosage after the reduction of arterial hypertension to stage 1 or below.^42^ In cases of an asymptomatic decrease in LVEF of 10% or more from baseline and below institutional lower limits of normal from pretreatment level, the dosage of BRAF inhibitors should not be modified, but MEK inhibitors should be stopped. A follow-up should be made after 4 weeks, and the therapy with MEK inhibitors should be resumed in the event of recovery of LVEF to normal values or permanently discontinued in the event of persistent changes in LVEF. In case of heart failure or a decrease in LVEF of 20% or more from baseline that is below the lower limit of normal, therapy with BRAF inhibitors should be stopped and, in the event of recovery, resumed at the same dosage, while therapy with MEK inhibitors should be permanently discontinued.^43^ Initiation of treatment with BRAF inhibitors is not recommended in patients with QTc of more than 500 milliseconds, and BRAF inhibitors should be stopped at a QTc of more than 500 milliseconds or at an increase in QTc of more than 60 milliseconds from baseline values at any time during the treatment. In case of increased QTc of less than 60 milliseconds from baseline, the BRAF inhibitors should be discontinued and reintroduced at a reduced dosage in the event of recovery. The measurement of QTc and serum electrolytes is recommended before treatment, after 1 month of treatment, and after any dosage modification made for prolonged QTc.^18,42^ There are no specific indications for the management of pulmonary embolism, myocardial infarction, or atrial fibrillation, and these should be managed according to the cardiology guidelines, with BRAF and MEK inhibitor treatment considered separately for every individual.

### Limitations

This study has some limitations that need to be addressed. First, the definition of adverse events is based on the oncological recommendations that are not perfectly aligned with cardiological definitions. Second, the double therapy regimen was compared with single therapy, and a perfect delimitation of the adverse events deriving from BRAF inhibitors or MEK inhibitors cannot be done. Third, the treatment regimens were different between the studies; although from the same class of therapies, there are some specific adverse events related to each regimen. Fourth, because this is a study-level meta-analysis, it is not possible to make inferences regarding which individual patients are at higher risk of CVAEs. Fifth, 1 study^17^ was analyzed as 2 separate studies, which could induce bias in the final analysis. However, excluding an arm from the final analysis did not influence the conclusion. Sixth, many studies did not report on CVAEs and could not be included in the final analysis.

## Conclusions

In conclusion, therapy with BRAF and MEK inhibitors was associated with an increased risk of CVAEs, especially pulmonary embolism, a decrease in LVEF, and arterial hypertension, compared with BRAF inhibitor monotherapy. The risks of myocardial infarction, atrial fibrillation, and QTc prolongation were similar between the assessed groups. These adverse events should be carefully approached in cardio-oncology teams for an optimal treatment of patients with melanoma.

## Notes

Supplement. **eFigure 1.** Overall and Individual Study Estimates of the RR of Pulmonary Embolism and Myocardial Infarction Associated With BRAF and MEK Inhibitor Treatment vs BRAF Inhibitor Monotherapy **eFigure 2. **Overall and Individual Study Estimates of the RR of Atrial Fibrillation and QTc Interval Prolongation Associated With BRAF and MEK Inhibitor Treatment vs BRAF Inhibitor Monotherapy **eFigure 3. **The Quality of the Included Studies as Analyzed per Cochrane Handbook Recommendation **eTable 1. **Search Strategy Through PubMed on November 30, 2018 **eTable 2.** Definition of the End Points According to the National Cancer Institute’s Common Terminology Criteria for Adverse Events Version 4 **eTable 3.** Study and Patient Characteristics **eReferences**
