# Supplementary material for: Cardiovascular Adverse Events Associated With BRAF and MEK Inhibitors: A Systematic Review and Meta-analysis
Source: JAMA Netw Open. 2019 Aug 9;2(8):e198890. doi: 10.1001/jamanetworkopen.2019.8890 (PMC6692687; doi:10.1001/jamanetworkopen.2019.8890)
Supplement: Supplement. — eFigure 1. Overall and Individual Study Estimates of the RR of Pulmonary Embolism and Myocardial Infarction Associated With BRAF and MEK Inhibitor Treatment vs BRAF Inhibitor Monotherapy eFigure 2. Overall and Individual Study Estimates of the RR of Atrial Fibrillation and QTc Interval Prolongation Associated With BRAF and MEK Inhibitor Treatment vs BRAF Inhibitor Monotherapy eFigure 3. The Quality of the Included Studies as Analyzed per Cochrane Handbook Recommendation eTable 1. Search Strategy Through PubMed on November 30, 2018 eTable 2. Definition of the End Points According to the National Cancer Institute’s Common Terminology Criteria for Adverse Events Version 4 eTable 3. Study and Patient Characteristics eReferences [file jamanetwopen-2-e198890-s001.pdf]

Supplementary Online Content

Mincu RI, Mahabadi AA, Michel L, et al. Cardiovascular adverse events associated with BRAF and MEK inhibitors: a systematic review and meta-analysis. *JAMA Netw Open*. 2019;2(8):e198890. doi:10.1001/jamanetworkopen.2019.8890

eFigure 1. Overall and Individual Study Estimates of the RR of Pulmonary Embolism and Myocardial Infarction Associated With BRAF and MEK Inhibitor Treatment vs BRAF Inhibitor Monotherapy

eFigure 2. Overall and Individual Study Estimates of the RR of Atrial Fibrillation and QTc Interval Prolongation Associated With BRAF and MEK Inhibitor Treatment vs BRAF Inhibitor Monotherapy

eFigure 3. The Quality of the Included Studies as Analyzed per Cochrane Handbook Recommendation

eTable 1. Search Strategy Through PubMed on November 30, 2018

eTable 2. Definition of the End Points According to the National Cancer Institute's Common Terminology Criteria for Adverse Events Version 4

eTable 3. Study and Patient Characteristics

eReferences

This supplementary material has been provided by the authors to give readers additional information about their work.

© 2019 Mincu RI et al. *JAMA Network Open.*

eFigure 1. Overall and Individual Study Estimates of the RR of Pulmonary Embolism and Myocardial Infarction Associated With BRAF and MEK Inhibitor Treatment vs BRAF Inhibitor Monotherapy

a.

Study name	MH risk ratio	Lower limit	Upper limit	Z-Value	p-Value
	Statistics for each study				
Ascierto[10] 2016	1,99	0,18	21,82	0,56	0,57
Dummer[17] E 2018	6,00	0,73	49,37	1,67	0,10
Dummer[17] V 2018	5,81	0,71	47,82	1,64	0,10
	4,36	1,23	15,45	2,28	0,02

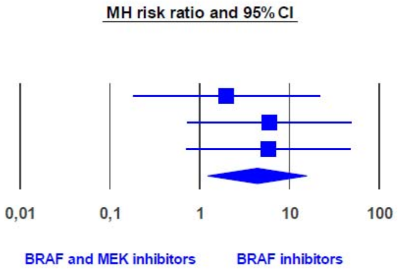

b.

Study name	MH risk ratio	Lower limit	Upper limit	Z-Value	p-Value
	Statistics for each study				
Ascierto[10] 2016	1,99	0,18	21,82	0,56	0,57
Dummer[17] E 2018	2,00	0,18	21,87	0,57	0,57
Dummer[17] V 2018	0,97	0,14	6,81	-0,03	0,97
	1,46	0,41	5,25	0,58	0,56

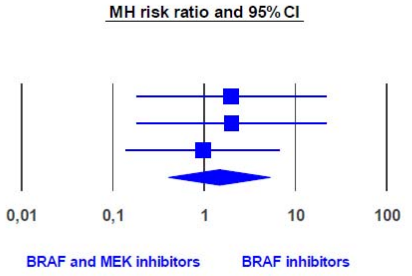

© 2019 Mincu RI et al. *JAMA Network Open.*

c.

Study name	MH risk ratio	Lower limit	Upper limit	Z-Value	p-Value
Ascierto[10] 2016	2,99	0,12	72,99	0,67	0,50
Dummer[17] E 2018	5,00	0,24	103,47	1,04	0,30
Dummer[17] V 2018	4,84	0,23	100,24	1,02	0,31
	4,22	0,71	24,99	1,58	0,11

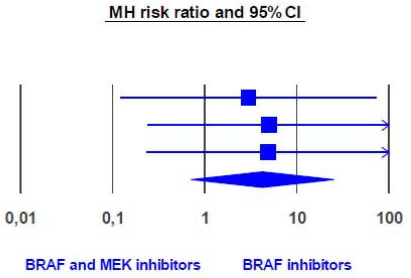

d.

Study name	MH risk ratio	Lower limit	Upper limit	Z-Value	p-Value
Ascierto[10] 2016	2,99	0,12	72,99	0,67	0,50
Dummer[17] E 2018	3,00	0,12	73,19	0,67	0,50
Dummer[17] V 2018	2,91	0,12	70,90	0,65	0,51
	2,96	0,47	18,75	1,15	0,25

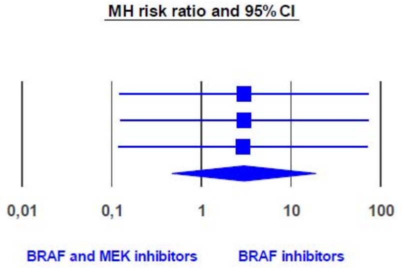

Legend: a. all-grade pulmonary embolism; b. high-grade pulmonary embolism; c. all-grade myocardial infarction; d. high-grade myocardial infarction; RR = risk ratio; BRAF = B-Raf proto-oncogene serine/threonine kinase; MEK = mitogen-activated protein kinase. Square boxes denote the RR for each study and parallelogram boxes denote the overall estimated RR, and horizontal lines represent 95% confidence intervals.

© 2019 Mincu RI et al. *JAMA Network Open.*

eFigure 2. Overall and Individual Study Estimates of the RR of Atrial Fibrillation and QTc Interval Prolongation Associated With BRAF and MEK Inhibitor Treatment vs BRAF Inhibitor Monotherapy

a.

Study name	Statistics for each study				
	MH risk ratio	Lower limit	Upper limit	Z-Value	p-Value
Ascierto[10] 2016	2,99	0,82	10,90	1,66	0,10
Dummer[17] E 2018	0,25	0,03	2,22	-1,25	0,21
Dummer[17] V 2018	0,32	0,03	3,08	-0,98	0,33
	0,76	0,13	4,39	-0,31	0,76

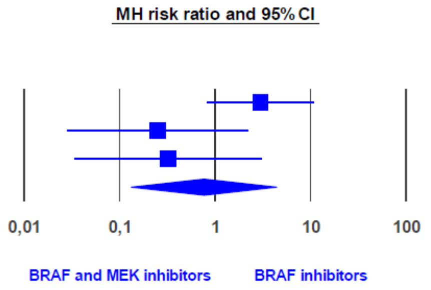

b.

Study name	Statistics for each study				
	MH risk ratio	Lower limit	Upper limit	Z-Value	p-Value
Ascierto[10] 2016	6,97	0,36	134,26	1,29	0,20
Dummer[17] E 2018	3,00	0,12	73,19	0,67	0,50
Dummer[17] V 2018	2,91	0,12	70,90	0,65	0,51
	4,05	0,67	24,40	1,53	0,13

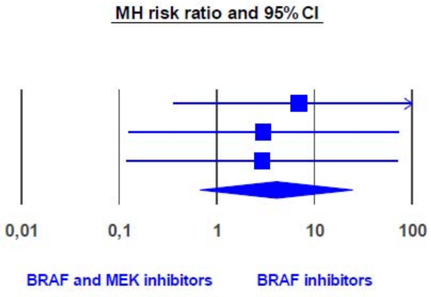

c.

Study name	Statistics for each study				
	MH risk ratio	Lower limit	Upper limit	Z-Value	p-Value
Ascierto[10] 2016	0,84	0,39	1,84	-0,43	0,67
Dummer[17] E 2018	0,07	0,00	1,16	-1,86	0,06
Dummer[17] V 2018	0,07	0,00	1,31	-1,77	0,08
	0,23	0,03	1,87	-1,37	0,17

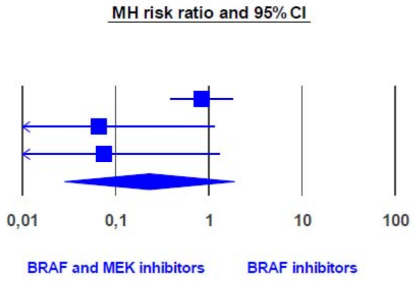

© 2019 Mincu RI et al. *JAMA Network Open.*

d.

Study name	Odds ratio	Lower limit	Upper limit	Z-Value	p-Value
Ascierto[10] 2016	1,00	0,20	4,98	-0,00	1,00
Dummer[17] E 2018	0,33	0,01	8,19	-0,67	0,50
	0,80	0,19	3,36	-0,31	0,76

(Columns 2–6: Statistics for each study)

Odds ratio and 95% CI

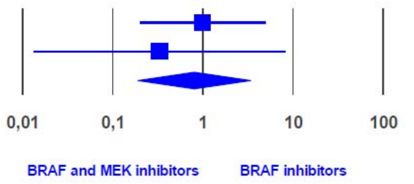

Legend: a. all-grade atrial fibrillation; b. high-grade atrial fibrillation; c. all-grade QTc interval prolongation; d. high-grade QTc interval prolongation; RR = risk ratio; BRAF = B-Raf proto-oncogene serine/threonine kinase; MEK = mitogen-activated protein kinase. Square boxes denote the RR for each study and parallelogram boxes denote the overall estimated RR, and horizontal lines represent 95% confidence intervals.

© 2019 Mincu RI et al. *JAMA Network Open.*

eFigure 3. The Quality of the Included Studies as Analyzed per Cochrane Handbook Recommendation

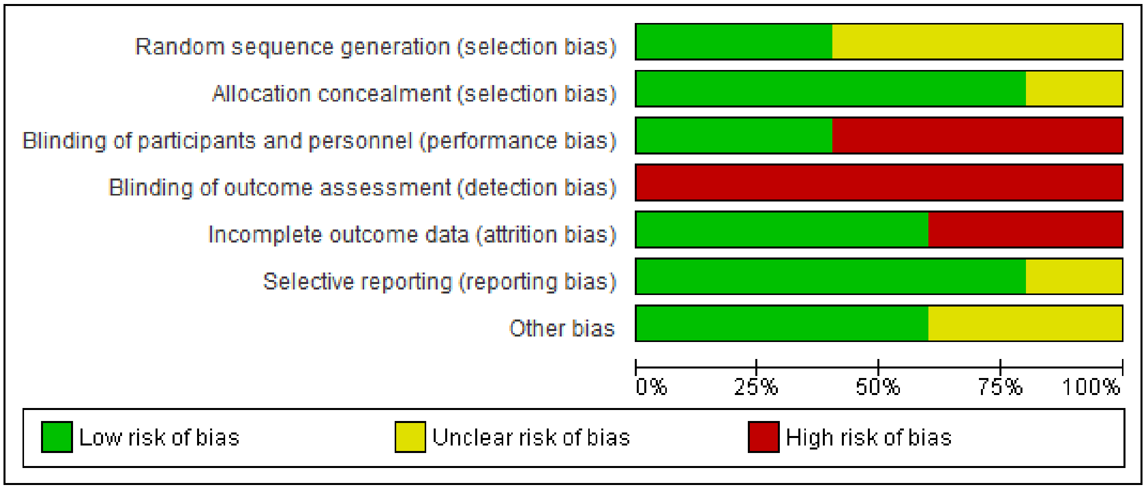

© 2019 Mincu RI et al. *JAMA Network Open.*

eTable 1. Search Strategy Through PubMed on November 30, 2018

Query	Pubmed Publications
Vemurafenib AND melanoma	1509
Dabrafenib AND melanoma	642
Encorafenib AND melanoma	34
Trametinib AND melanoma	472
Binimetinib AND melanoma	57
Cobinimetinib AND melanoma	116
Total	2830

© 2019 Mincu RI et al. *JAMA Network Open.*

eTable 2. Definition of the End Points According to the National Cancer Institute's Common Terminology Criteria for Adverse Events Version 4.[1]

High-grade endpoints are defined as grade ≥ 3.

**Endpoint definition**	**Grade 1**	**Grade 2**	**Grade 3**	**Grade 4**	**Grade 5**
Arterial hypertension was defined as a repeatedly elevation in the blood pressure (BP) exceeding 140 over 90 mm Hg.	prehypertension (systolic BP 120 - 139 mm Hg or diastolic BP 80 - 89 mm Hg)	stage 1 hypertension (systolic BP 140 - 159 mm Hg or diastolic BP 90 - 99 mm Hg); medical intervention indicated; recurrent or persistent (≥24 hrs); symptomatic increase by >20 mm Hg (diastolic) or to >140/90 mm Hg if previously WNL; monotherapy indicated Pediatric: recurrent or persistent (≥24 hrs) BP >ULN; monotherapy indicated	stage 2 hypertension (systolic BP ≥ 160 mm Hg or diastolic BP ≥ 100 mm Hg); medical intervention indicated; more than one drug or more intensive therapy than previously used indicated	life-threatening consequences (e.g., malignant hypertension, transient or permanent neurologic deficit, hypertensive crisis); urgent intervention indicated.	death

© 2019 Mincu RI et al. *JAMA Network Open.*

Decrease in the ejection fraction is defined as the drop-in percentage computed when the amount of blood ejected during a ventricular contraction of the heart is compared to the amount that was present prior to the contraction.	-	resting ejection fraction (EF) 50 - 40%; 10 - 19% drop from baseline	resting ejection fraction (EF) 39 - 20%; >20% drop from baseline	resting ejection fraction (EF) <20%	-
Pulmonary embolism was defined as partial or total occlusion of the pulmonary artery and branches by thrombosis.	intervention not indicated.	minimal, local or noninvasive intervention indicated	medical intervention indicated	hemodynamic or neurologic instability; urgent intervention indicated	death
Atrial fibrillation is defined as a disorder characterized by a dysrhythmia without discernible P waves and an irregular ventricular response due to multiple reentry circuits. The rhythm disturbance originates above the ventricles.	asymptomatic, intervention not indicated	non-urgent medical intervention indicated	symptomatic and incompletely controlled medically, or controlled with device (e.g., pacemaker), or ablation	life-threatening consequences; urgent intervention indicated	death

© 2019 Mincu RI et al. *JAMA Network Open.*

Prolongation of QTc interval is defined as a finding of a cardiac dysrhythmia characterized by an abnormally long corrected QT interval.	QTc 450 - 480 ms	QTc 481 - 500 ms	QTc ≥ 501 ms on at least two separate ECGs	QTc >= 501 or >60 ms change from baseline and Torsade de pointes or polymorphic ventricular tachycardia or signs/symptoms of serious arrhythmia	-
Myocardial infarction is defined as a disorder characterized by gross necrosis of the myocardium; this is due to an interruption of blood supply to the area.	-	asymptomatic and cardiac enzymes minimally abnormal and no evidence of ischemic ECG changes	severe symptoms; cardiac enzymes abnormal; hemodynamically stable; ECG changes consistent with infarction	life-threatening consequences; hemodynamically unstable	death

© 2019 Mincu RI et al. *JAMA Network Open.*

eTable 3. Study and Patient Characteristics

Study Characteristic	Studies, No.	Patients, No
Total	5	2317
Phase		
2	1	108
3	4	2209
Median age, y		
≤ 55	2	812
> 55	3	1505
Melanoma status		
Stage ≥ IIIB unreseactable	5	2317
Regimen		
Dabrafenib +Trametanib vs. Dabrafenib	2	531
Dabrafenib +Trametanib vs. Vemurafenib	1	704
Vemurafenib+Cobimetinib vs. Vemurafenib or Encorafenib	1	495
Encorafenib+Binimetinib vs. Vemurafenib	1	577
Median follow-up		
≤ 15 Months	3	1307
> 15 Months	2	1010

© 2019 Mincu RI et al. *JAMA Network Open.*

eReferences

1. National Cancer Institute's Common Toxicity Criteria (version 4) 2009; https://evs.nci.nih.gov/ftp1/CTCAE/CTCAE_4.03_2010-06-14_QuickReference_5x7.pdf.

© 2019 Mincu RI et al. *JAMA Network Open.*
